# Re-irradiation for locally recurrent refractory breast cancer

**DOI:** 10.18632/oncotarget.6036

**Published:** 2015-10-08

**Authors:** Tomas Merino, William T. Tran, Gregory J. Czarnota

**Affiliations:** ^1^ Department of Radiation Oncology, Sunnybrook Health Sciences Centre, Toronto, Canada; ^2^ Department of Radiation Oncology, Faculty of Medicine, University of Toronto, Toronto, Canada; ^3^ Departamento de Hemato-Oncologia, Pontificia Universidad Catolica de Chile, Santiago, Chile

**Keywords:** breast cancer, radiotherapy, re-irradiation, recurrent, refractory breast cancer

## Abstract

**Purpose:**

To report an analysis of treatment outcomes of a cohort of patients re-irradiated for locally recurrent refractory breast cancer (LRRBC)

**Patients and Methods:**

Between 2008 and 2013, 47 women (mean age = 60 years) were re-irradiated for LRRBC. Outcomes were measured using Kaplan-Meier log rank to compare curves and Cox regression for multivariate analysis. Outcomes included overall survival (OS), time to re-treatment, survival without systemic progression, and survival without local recurrence.

**Results:**

Fifty-six instances of re-irradiation were completed and analyzed. The mean cumulative 2 Gy equivalent dose (EQD2) to the whole breast and tumour cavity (α/β = 3) was 99.8 Gy and 109.1 Gy, respectively. Most patients initially had significant symptoms before RT due to local recurrence. The median time to re-treatment and to systemic failure was 41 and 50 months, respectively. Median follow-up for OS was 17 months and OS was 0.73 (SE = 0.07) at 1 year and 0.67 (SE = 0.07) at 2 years. Local control was 0.62 (SE = 0.07) and 0.5 (0.08) at 1 and 2 years, respectively. Acute radiation dermatitis was G1-2, G3 and G4 in 45, 4 and 1 cases, respectively. One patient presented with necrosis. The most common long term toxicity was G3 fibrosis (*n* = 4) and telangiectatic changes (*n* = 3). Multivariable analysis indicated that skin involvement (Hazard Ratio = 6.6 (1.4-31), *p* = 0.016) and time to local recurrence <2yr (HR 3.1 (1.04-9.7) *p* = 0.042) predicted local recurrence.

**Conclusion:**

High dose re-irradiation is feasible for locally RRBC. This approach can have a significant benefit in this very high-risk group.

## INTRODUCTION

Breast cancer is the most frequently diagnosed cancer in North American women. In 2014, in Canada it is estimated that approximately 24,400 new cases and about 5000 deaths will occur as a result of the disease [1]. Since 2004 the incidence of breast cancer has stabilized but breast cancer mortality rates have declined due to improved mammography screening [2] and the use of more effective therapies following breast cancer surgery [3, 4]. Local recurrence rates are approximately 10-20% at 10 and 15 years respectively, but can be as high as 40% depending on treatment, patient age, and primary tumour size [5, 6]. Local recurrence is often a painful situation for breast cancer patients since many patients, even those with metastatic disease, may still live many months to years with appropriate treatment [7-9]. Uncontrolled locally recurrent breast cancer can cause many significant problems that can decrease the quality of life of patients. These problems include ulcerations, bleeding, arm edema, pain and brachial-plexus palsy. One study found that 62% of patients had one or more of the above problems due to uncontrolled loco-regional recurrent breast cancer [10]. Treatment options may involve systemic and surgical intervention, as well as radiotherapy. Standard treatment for local recurrence after breast-conserving surgery and radiotherapy involves total mastectomy. In circumstances where recurrences occur after mastectomy, the prognosis is relatively poor with a survival of 46-55% at 5 years and 28% at 10 years [11-13]. Post-operative radiotherapy has been shown to play a beneficial role in the primary setting with effective local control and improved overall survival [14, 15]. However, proposals to administer second radiotherapy treatment have been met with resistance and major concerns regarding side effects have limited the adoption of re-irradiation for breast cancer. Previously irradiated treatment sites are a therapeutic challenge, particularly since there is limited data about the curative potential of radiation in this context. Additionally, wide-spread hesitation to implement re-irradiation treatments is based on limited data about the potential radiobiological effects and damage. A previous study examined re-irradiated head and neck carcinoma and reported mucosal necrosis in 21% of patients and trismus in 30% of patients [16]. However, other research on breast cancer has indicated mild to moderate side effects from re-irradiation [17-19]. Hannoun-Levi *et al.* [20] presented the results of 217 breast cancer patients retreated with a multi-catheter approach for an isolated local recurrence. The most common toxicity was subcutaneous fibrosis and telangiectasia, grades 1, 2, 3 and 4 in 50%, 39%, 10% and 1%, respectively. Resch et al. re-treated 17 breast cancer patients with either brachytherapy or external beam radiation [21]. All patients had grade 1-2 fibrosis and two patients developed minimal telangiectasia. Harms et al. treated 58 breast cancer patients, of whom less than 10% had grade 4 toxicity [22]. These studies suggest that a second full radiation treatment may be feasible without severe side effects. Other studies have also found good local control from re-irradiation to local breast cancer recurrences. Linthorst [23] used external beam radiation (32 Gy) and hyperthermia to treat recurrent breast cancer with a 78% local control at five years. Kauer-Dorner published the results of partial breast re-irradiation with pulse dose rate (PDR) brachytherapy with follow-up (FU) at 37 months and a 5 year local control of 93% [19]. Hannoun-Levi published the results of re-irradiation with a multicatheter brachytherapy approach where local recurrence was 5.7% and 7.2% at 5 and 10 years respectively [20]. In contrast, a recent publication of the CALOR (chemotherapy for isolated locoregional recurrence of breast cancer) trial has established the role of chemotherapy for patients with isolated local recurrent breast cancer with a benefit of 12% increasing overall survival to 69% vs. 57% at five years [24].

Although emerging studies are recognizing the potential responsiveness of recurrent breast cancer to treatment, more data is required to understand the therapeutic benefits of re-irradiation for survival and its toxicity. In the present study, we determined the safety and efficacy of radiation therapy for patients who developed local recurrences following previous radiation treatment. The aim of this study is to report the survival and local control outcomes of patients treated with re-irradiation and find clinical or pathological prognostic factors for survival and local failure in this group of patients. In short, patients (n=47) received a mean cumulative 2 Gy equivalent dose (EQD2) to the whole breast and tumour cavity (α/β=3) of 99.8 Gy and 109.1 Gy, respectively. The median time to re-treatment and to systemic failure was 41 and 50 months, respectively. Overall survival was 0.73 (SE=0.07) at 1 year and 0.67 (SE=0.07) at 2 years. Local control was 0.62 (SE=0.07) and 0.5 (0.08) at 1 and 2 years, respectively.

## RESULTS

Patients' characteristics indicated that this group was at high risk for local and systemic recurrence: 97% of patients were grade 2 or 3, 46% ER negative, 65% PR negative, 28% Her2 positive, 60% LVI positive, and 61% with axillary involvement. The median time to local recurrence was 41 months. Local recurrence location was on the chest wall (n=18), breast same quadrant (n=8), breast non-specified (n=7), supraclavicular area (n=6), axilla (n=5), breast other quadrant (n=4), and sternum (n=2). For recurrence, 18 patients had further surgery, 11 had mastectomy and 7 had local excision before re-irradiation. An important number of patients had significant symptoms from the local progression including pain, bleeding bad odor and swelling. Seventy three percent of patients had gross macroscopic disease at the time of re-irradiation.

**Figure 1:**
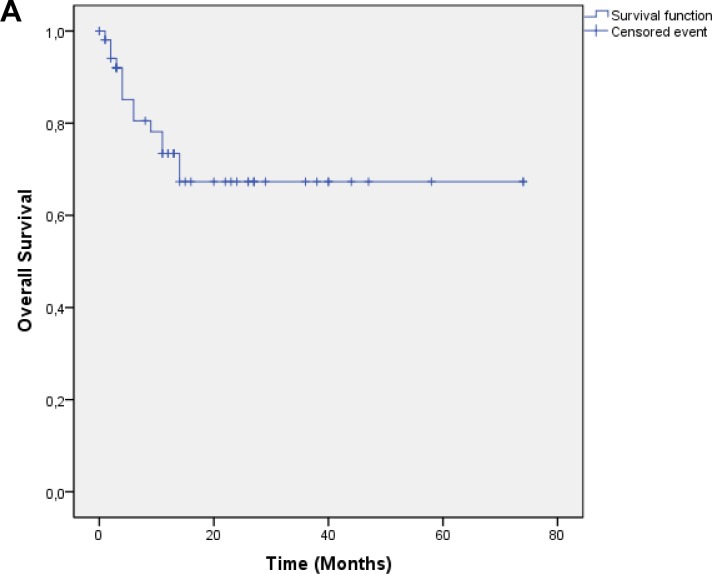

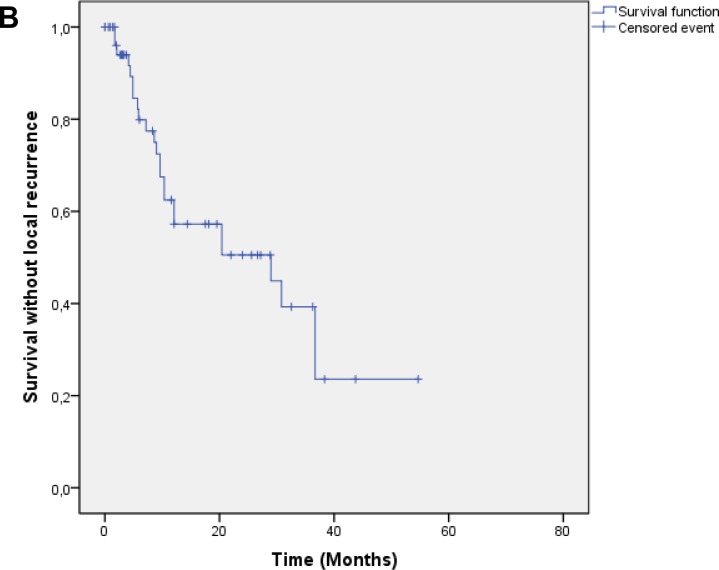
A: Overall Survival B: Survival without local recurrence

**Figure 2:**
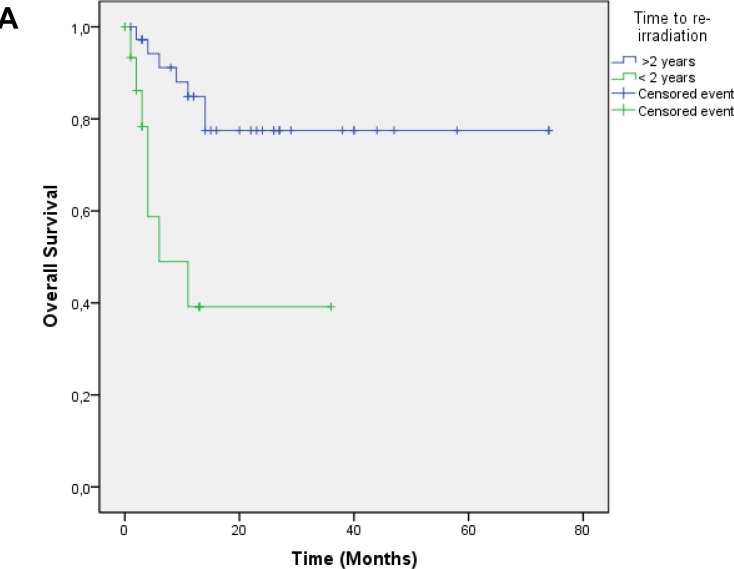

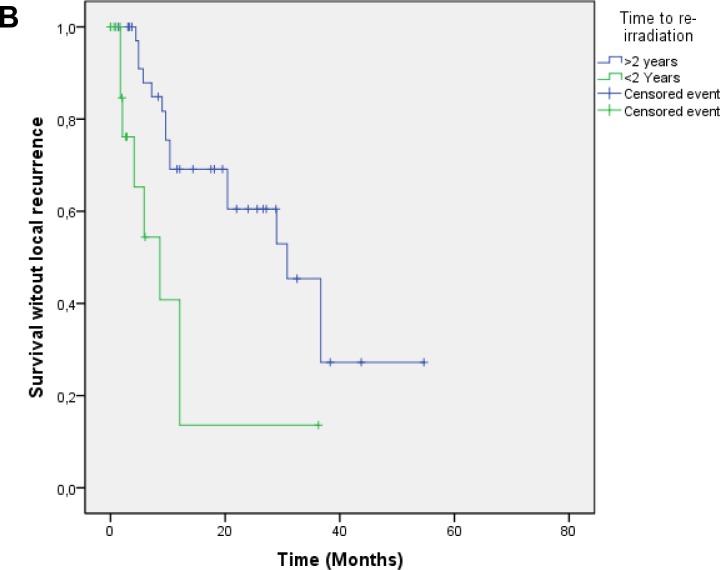
A: Overall survival according to time to re-irradiation Footnote: > 2 years to re-irradiation (blue), < 2 years to re-irradiation (green), ***p*** = 0.001. B: Survival without local recurrence according to time to re-irradiation. Footnote: > 2 years to re-irradiation (blue), < 2 years to re-irradiation (green), ***p***= 0.002.

**Figure 3:**
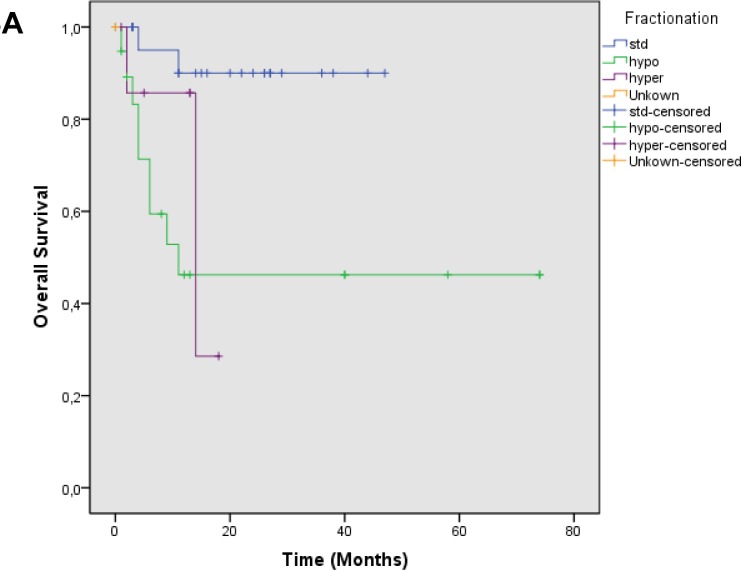

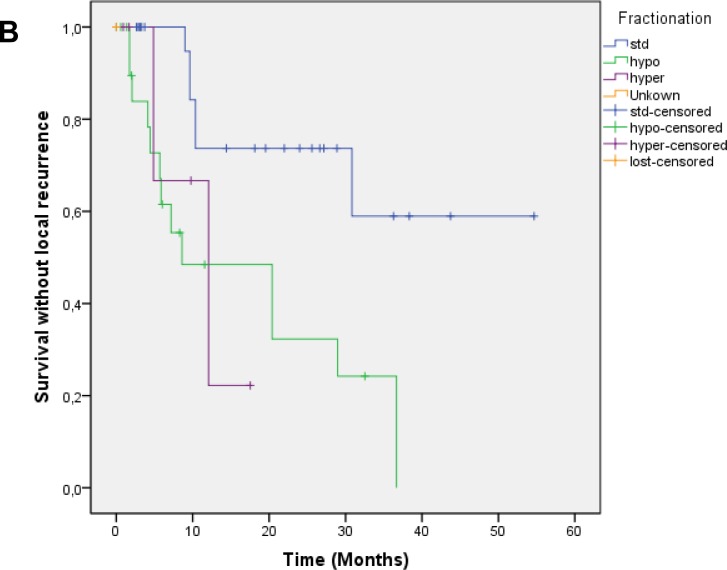
A: Overall survival according to fractionation scheme Footnote: Standard Fractionation (blue), Hypofractionation (green), Hyperfractionation (purple), Unkown (orange), p=0.002 Standard vs Hypofractionation, p= 0.02 Standard vs Hyperfractionation p=0.59 Hypofractionation vs hyperfractionation. B; Survival without local recurrence according to fractionation scheme. Footnote: Standard Fractionation (blue), Hypofractionation (green), Hyperfractionation (purple), Unkown (orange), *p* = 0.01 Standard vs Hypofractionation, *p* = 0.04 Standard vs Hyperfractionation p=0.87 Hypofractionation vs hyperfractionation

Median follow up was 17.4 months, OS was 0.73 at 1 year and 0.67 at 2 years [Figure 1a]. Local control was 0.63 at 1 year and 0.50 at 2 years. Median time to local failure was 28.9 months [Figure 1b]. Survival without systemic failure was analyzed from the original treatment with a median FU of 78.6 months. Survival without systemic failure was 49% at 5 years. [Supplementary Figure 1]. Exploratory analysis was done for OS according to time to retreatment (< 2years). There was a significant difference in 1 year survival (0.85 vs. 0.39, p=0.001) favoring patients treated more than 2 years from the original treatment [Figure 2a]. Local control was 0.69 for patients with more than 2 years interval and 0.13 for those with less p=0.002 [Figure 2b]. Patients treated with standard fractionation had significantly better 1 year OS (p=0.002) and SWLR (p=0.01) than patients treated with hypofractionation and hyperfractionation (OS 0.90, 0.46 and 0.85. SWLR 0.73, 0.48 and 0.22 for standard,hypofractionation and hyperfractionation, respectively) [Figure 3a and 3b]. Skin involvement and BCS also showed a significant impact on OS (p=0.013 and p=0.043, respectively) [Supplementary Figure 2a and 2b]. Local control showed no significant difference in OS [Supplementary Figure 3]. ER status, margin status (macroscopic disease) showed no impact on OS or SWLR. [Supplementary Figure 4a and 4b] The surgery performed had no impact on SWLR [Supplementary Figure 5]. Multivariable analysis suggested that OS was better for standard fractionation than for hypofractionation HR 7.8 (1.2-51.7) and hyperfractionation 10.9 (1.4-86.8), p=0.032 and 0.23, respectively. SWLR was worse for hypofractionation compared to standard fractionation (HR 5.3(1.2-22.7)) but not significantly different for hyperfractionation (HR 6.7(0.6-72.5)).

**Figure 4:**
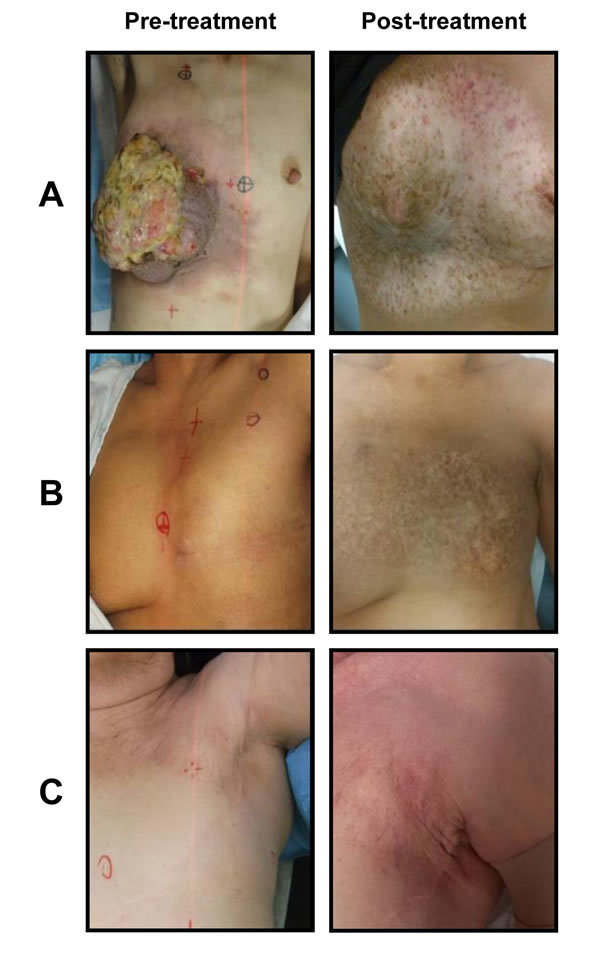
Treatment effects after whole chest wall/breast + nodal fields irradiation Footnote: Three cases presented (Figures 4A, 4B, 4C). Patient A was originally treated for right sided invasive ductal carcinoma. 31 years later presented with triple negative un-resectable fungating mass. She was treated with re-irradiation with concomitant weekly cisplatin 20 mg/m^2.^She received 65 Gy in 25 fx BID to the breast + lymph node area and then a boost of 16 Gy in 8 fractions. Eighteen months post-treatment image shows significant fibrosis and telangiectasia but no residual disease. Patient B was originally treated with a mastectomy for an invasive ductal carcinoma and adjuvant radiotherapy (50 Gy in 25 fx) to the chest wall and lymph node area. 24 months after developed a 6.0 cm recurrence on the chest wall. She received 65 Gy in 25 fx BID. Postreatment image shows significant hyperpigmentation but no evidence of recurrence. Patient C was treated with a lumpectomy and adjuvant radiotherapy to the breast 42.5 Gy in 16 fx. 67 months after, she developed an axillary recurrence. She had re-irradiation to the chest wall and axilla 50 Gy in 25 fx and a boost of 16 Gy to the recurrent tumour. Image postreatment show some telangiectasia and fibrosis but no disease recurrence.

The most common acute toxicity was radiation dermatitis (n=44, G1-G2); 4 patients had G3 and 1 patient G4. Long term toxicity included fibrosis (n=4, G3) and telangiectasia G3 in 3 patients. One patient developed skin necrosis that healed after 3 months with medical treatment [Table 4]. Only one patient did not complete retreatment due to acute toxicity. Figure 4 presents representative images of patients before and after re-irradiation.

## DISCUSSION

To our knowledge, this is the first study to report the results of local re-irradiation for recurrent refractory breast cancer using external beam radiotherapy. We have demonstrated the feasibility of a systematic aggressive approach including systemic and local treatment in a population that generally demonstrates unfavorable outcomes. It is with such patients where radiotherapy is often unfortunately avoided or given to a low dose for palliation only due to the belief that the chest wall as a site of disease cannot be re-irradiated. The evidence here supports aggressive re-irradiation with good results for local control and survival. The prevalence of symptomatic disease in over half of the patients even after systemic treatments highlights the importance of local treatment and the aggressiveness of the disease. Over 70% of patients had clinical and symptomatic responses to radiation although a significant number developed subsequent progression in the field accounting for the 50% local control at 2 years. Most of these patients subsequently had further systemic treatment explaining their fairly good survival (67%) at 2 years. Our results confirm that radiotherapy can control locally recurrent disease even in the case of progression with chemotherapy with a 50% control rate at 2 years. Exclusive radiotherapy without surgery has been previously studied in the context of metastatic breast cancer by Bourgier [25] reporting 85% local control.

Our subgroup analysis provided interesting results, when analyzied according to tumor factors there was no significant difference in overall survival or local control according to ER status or the presence of macroscopic disease at the moment of re-irradiation. However, the presence of skin involvement and an interval of less than two years from the original RT to the re-irradiation course were significantly associated with worse OS and local control. In regards to the subgroup analysis by treatment factors there was a significant better local control and OS for those patients treated with a conventional fractionation compared to those treated with hypofractionation. This could potentially be explained as patients with a worse performance status could have been offered a shorter treatment but other explanations including an increased sensitivity to standard fractionation in this context cannot be ruled out.

Although better OS was seen in the group of patients treated with BCS compared to those treated with a mastectomy, the absence of a difference in local control when analyzing the same variable suggests a selection bias favoring BCS for the most favorable patients. Although the effect of local regional treatment on overall survival in the metastatic setting is a matter of active discussion [26-28] with some retrospective data suggesting a benefit [29-31], two randomized controlled trials recently presented showed no improvement in survival for patients undergoing loco-regional treatment (SABCS 2013;Badwe R: Abstract S2–02, Soran, A: Abstract S2-03). The primary goal of any loco-regional treatment is to avoid local progression and reduce disease burden and through local control, improve the quality of life of patients even when no survival benefit has been proven yet.

Re-irradiation in the present study demonstrated very good results and acceptable toxicity. Furthermore, although difficult to compare with other series, the results here in terms of side effects demonstrate a similar profile to what has been previously reported and were not prohibitive of retreatment to a full potentially curative dose.

Kauer-Dorner [19] followed 39 patients after re-irradiation with pulse dose radiotherapy and the most common morbidity was fibrosis 83% (4% presented with grade 3), telangiectasia (32%) and hyperpigmentation (29%). Hannoun-Levi [20] also presented the results of re-irradiation with a partial breast multicatheter, where fibrosis was present in 67%, telangiectasia in 16%, and hyperpigmentation in 9% of treated patients. Only 10% and 1% of patients presented with grade 3 and grade 4 long term effects. Oldenborg [32] used a different approach with re-irradiation and hyperthermia for seventy five recurrent breast cancer patients with eight and seven patients developing grade 3 telangiectasia and fibrosis, six patients developed G4 ulceration.

In the series here after high dose re-irradiation, only about half of the patients developed clinical fibrosis and 1/3 of these were grade 3, approximately 1/4 of the patients studied developed telangiectasia, half of which were grade 3, and only one patient developed ulceration. These numbers could be lower due to the smaller dose per fraction and absence of brachytherapy and hyperthermia in this series but could also be due to different grading scales or differences in follow up and should be interpreted with caution.

Limitations of the study here include the number of patients examined, and relatively short follow-up periods. Also variable patient characteristics and different radiation schemes used could have affected the results. Despite these limitations our data suggest that re-irradiation to a full dose is feasible and can provide long-term local control with symptomatic benefit. The benefits outweigh the risks of retreatment and offer control to patients who would otherwise not be treated or given a low palliative dose only.

## CONCLUSIONS

High dose re-irradiation is feasible for locally recurrent refractory breast cancer.

A significant number of patients never receive or are referred late for re-irradiation for fear of adverse effects and are unnecessarily exposed to local progression symptoms. An early multidisciplinary management approach for local recurrence considering surgery, systemic management and re-irradiation can have a significant benefit to this very high-risk group. The work here forms a basis for administering re-irradiatuion to the breast or chest wall for breast cancer recurrence.

## MATERIALS AND METHODS

### Patients and treatment

This retrospective study was institutional review board approved. Electronic medical charts were used to collect data on patients treated from January 2008 to January 2013 for recurrent breast cancer. 56 Re-irradiations were performed and analyzed. Specifically, forty-seven patients were treated with re-irradiation, two patients were treated bilaterally and 7 had an ipsilateral recurrence and had a second re-irradiation course.

Patients' characteristics at the time of original therapy and re-treatment are provided in Table 1. The detailed characteristics of the patients and treatment at the original and re-irradiation are detailed in Supplementary Table 1 and Table 2. The subgroup of patients that had a second re-irradiation are shown in Supplementary Table 2.

**Table 1:** Patient, tumour and treatments characteristics

Characteristic		Primary		Recurrence	
Patient		(n)	Median (range) / %	(n)	Median (range) / %
Age (years)			54(23-83)		60(30-89)
Time to recurrence (months)					41
pT size cm			4.3 (0.3-19)		6.5(0.5-22)
Grade	1	1	2.9	3	10.7
	2	13	38.2	5	17.8
	3	20	58.8	20	71.4
	Unknown	15		30	
Estrogen R	Positive	24	53	21	63.6
	Negative	21	46	12	36.4
	Unknown	4		16	
Progesterone R	Positive	13	34	12	46.1
	Negative	25	65	14	53.8
	Unknown	11		23	
Her2 Status	Positive	8	26	1	3.3
	Negative	22	73	29	96.6
	Unknown	19		19	
LVI	Positive	17	60	5	45.4
	Negative	11	39	6	54.5
	Unknown	21		38	
LN	Positive	26	61	12	66.6
	Negative	16	38	6	33.3
	Unknown	7		31	
Skin involved	Positive	8	25	15	53.5
	Negative	24	75	13	46.4
	Unknown	17		21	
Margin	Macro +	5	17	32	72.7
	Micro +	4	14	3	6.8
	Negative	19	67	9	20.4
	Unknown	21		5	
Treatment					
Surgery	Mastectomy	22	46	11	22.4
	Lumpectomy	24	50	7	14.2
	None	3	4	31	63.2
Axilar Dissection	Yes	15	37	5	11.6
	No	26	63	38	88.3
	Unknown	8		6	
SLN Dissection	Yes	21	53	3	6.8
	No	19	47	41	93.1
	Unknown	9		5	
Hormonal Treat.*	Yes	23	58	20	57.1
	No	17	42	15	42.8
	Unknown	7		12	
Chemotherapy*	Yes	34	68	34	79.0
	No	7	32	8	18.6
	Unknown	6		5	
Radiotherapy	Breast/Chest Wall	41		48	
	Partial breast	0		8	
	Axila/SCV	24		16	
	Dose (Gy)		50.0		50.0
	Std fx	35	50.0	24	50.0
	Hyperfx	3	65.0	6	65.0
	Hypofx	9	42.5	18	42.5
	Unkown	2		1	
	Boost	21		17	
	WBRT Dose EQD2 α/β=10	48.3		96.7	
	WBRT Dose EQD2 α/β=3	48.7		99.8	
	Tu Cavity Dose EQD2 α/β= 10	55.3		105.9	
	Tu Cavity Dose EqD2 α/β =3	55.8		109.1	

SCV: Supraclavicular, LN: lymphnode, LVI: lymphvascular invasion, SLN: Sentinel lymphnode, WBRT: whole breast radiation therapy

* n=47 (2 patients had bilateral treatment)

**Table 2:** Patient characteristics at re-treatment

Pt	Age (years)	Time to retreatment (months)	Surgery	Tumour Size (cm)	ER	LN status	Total dose WBRT (Gy) EQD2 α/β = 3	Total dose WBRT (cGy) EQD2 α/β = 10
1	69	86	No	Unknown	(−)	Unknown	122.0	115.0
2*	70	26	LE	1.0	(−)	(+)	122.0	115.0
3	71	277	MRM	4.0	(+)	(−)	125.0	122.1
4	62	8	No	2.0	Unknown	Unknown	93.5	92.0
5	84	250	BCS	3.8	(+)	Unknown	Unknown	Unknown
6	55	20	LE	15.0	Unknown	Unknown	122.0	112.6
7	80	55	No	1.5	Unknown	Unknown	116.0	116.0
8	49	16	No	17.0	(+)	Unknown	90.7	94.1
9	71	181	BCS	1.3	(+)	Unknown	Unknown	Unknown
10	65	136	No	7.4	(+)	(+)	120.0	120.0
11	57	138	No	Unknown	(+)	Unknown	127.6	120.3
12	50	163	Unknown	7.5	Unknown	Unknown	93.2	89.0
13	72	138	LE	5.0	(+)	Unknown	83.0	75.4
14	54	49	MRM	4.4	(+)	(−)	124.1	120.8
15	84	118	LE	0.6	(+)	Unknown	110.0	110.0
16	44	35	No	8.5	Unknown	Unknown	121.8	117.8
17	51	49	No	1.2	Unknown	(+)	93.2	94.2
18	43	41	No	Unknown	(+)	(+)	102.0	98.5
19	64	69	MRM	1.3	(−)	(−)	116.0	116.0
20	67	29	LE	1.3	Unknown	(+)	122.7	124.6
21	68	67	MRM	2.0	(+)	(+)	98.1	94.8
22	81	33	No	3.5	Unknown	(−)	122.8	110.7
23	89	76	No	2.0	(−)	(+)	114.1	110.8
24	44	10	No	8.0	Unknown	(+)	117.5	104.4
25	59	30	No	3.5	Unknown	Unknown	118.1	114.8
26	34	17	BCS	1.5	(−)	(−)	116.0	116.0
27	43	33	No	1.1	(+)	(+)	132.8	132.4
28	58	25	No	Unknown	(−)	(+)	109.2	110.3
29	36	21	No	2.1	(−)	Unknown	109.9	109.9
30	82	27	No	10.0	(−)	Unknown	106.0	96.7
31	73	157	Unknown	4.3	(+)	(+)	127.8	123.8
32	30	29	LE	Unknown	(+)	Unknown	116.0	116.0
33	51	21	LE	3.0	(−)	Unknown	110.9	113.5
34	65	149	No	Unknown	(−)	(+)	116.0	114.3
35	55	6	No	Unknown	Unknown	Unknown	126.0	116.7
36	67	95	No	Unknown	(+)	Unknown	114.0	116.0
37*	69	12	No	Unknown	Unknown	(+)	83.4	87.3
38	48	14	No	Unknown	(−)	Unknown	132.0	132.0
39	45	8	No	22.0	(+)	Unknown	105.9	111.2
40	66	24	No	6.0	Unknown	Unknown	105.9	111.2
41	60	12	BCS	1.0	Unknown	Unknown	117.8	104.5
42	64	212	No	6.5	(+)	Unknown	131.8	107.4
43	49	218	BCS	0.5	(+)	(−)	Unknown	Unknown
44	63	3	No	12.0	Unknown	Unknown	76.4*	54.0*
45	56	154	No	10.6	(+)	Unknown	Unknown	Unknown
46	48	107	MRM	21.0	(+)	Unknown	132.0	132.0
47	54	371	No	15.0	(−)	Unknown	131.9	137.2
48	61	12	No	Unknown	Unknown	Unknown	116.1	114.8
49	41	48	LE	1.0	(+)	Unknown	Unknown	Unknown

BCS: breast conserving surgery, ER: Estrogen receptor, LE: local excision, MRM modified radical mastectomy, WBRT: whole breast radiotherapy.

* Patient 44 suspended radiotherapy after 6 Gy had recurrence and then full dose re-irradiation. * Patient 2 corresponds to contralateral re-irradiation of patient 1, Patient 37 corresponds to contralateral re-irradition of patient 36

Patients included in this study were referred for adjuvant radiotherapy treatment that involved definitive or aggressive palliative re-irradiation to the breast/chest wall +/− regional lymph nodes. All patients had pathologically confirmed recurrent breast cancer and had been previously treated with radiation to the breast/chest wall and were retreated to the same area. Twenty patients had a chest wall relapse after a mastectomy (MTT) and had re-irradiation to the ipsilateral chest wall, twenty one patients had a ipsilateral breast recurrence, and in this group 7 had a MTT and then chest wall re-irradiation. Four patients had a second lumpectomy and breast re-irradiation, and ten patients were non-ressectable and had re-irradiation without further surgery. Four patients had an isolated axillary recurrence, six had an isolated supraclavicular recurrence, three patients had a sternal mass recurrence, one patient had a chest wall and axillary recurrence and one had axilary and supraclavicular recurrence.

Patient and tumour characteristics were recorded in regards to the original and re-irradiation treatment including age at diagnosis, tumour size, hormonal status, grade, histology, disease margins, lymphovascular involvement (LVI), skin involvement, and lymph node status and survival [Tables 1, 2 and Supplementary Table 1].

Most of the patients had systemic treatment including 23 patients with hormonal therapy and 34 with chemotherapy; the most common chemotherapy schemes used included Adriamycin Cyclosphosphamide Taxol (ACT), 5-Fluoroucilracil Epirubicin Cyclosphosphamide-Docetaxel (FEC-D), and Capecitabine, Taxotere, Cyclosphosphamide Metrotrexate Fluoracil (CMF). Six patients had concomitant chemoradiotherapy with weekly cisplatin. Details of patient's systemic treatments are provided in Supplementary Table 3.

Initial therapy followed institutional guidelines at Sunnybrook Health Sciences Centre, Toronto, Canada and involved a combination of systemic therapy, surgical resection (mastectomy or lumpectomy) and radiotherapy.

The symptoms from recurrent disease at the time of the retreatment included: bleeding, pain, ulceration of the skin, bad odour, lymphedema and brachial plexus dysfunction and are presented in Table 3. A significant group (n=33) had local progression under systemic chemotherapy for oligometastasic disease.

**Table 3:** Patients symptoms before treatment and symptomatic response

Symptom before Re-irradiation	Number of Patients	Improvement
Pain	24	15
Ulceration	14	8
Bleeding	9	3
Brachial plexus involvement	9	8
Lymphedema	7	5
Bad odour	4	2
No symptoms from recurrence	28	
One symptom	8	6
Two symptoms	6	5
Multiple symptoms	14	9

Corresponds to symptoms at re-irradiation

**Table 4:** Acute and late toxicity

Acute toxicity (Total)		G0	G1-G2	G3	G4	Not Available
	Radiation dermatitis	0	44	4	1	7
By Subgroup	Gross disease	0	23	5	0	4
	No gross disease	0	13	3	0	0
	Mastectomy	0	25	4	0	4
	BCS	0	15	2	0	0
	Hypofractionation	0	10	4	0	4
	Standard fractionation	0	22	2	0	0
Late toxicity (Total)						
	Fibrosis	19	11	4	0	15
	Telangiectasia	27	4	3	0	15
	Necrosis	33	0	0	1	15
	Lymphedema	30	4	0	0	15
	Hyper/Hypopigmentation	28	6	0	0	15
	Neumonitis	32	2	0	0	15
By Subgroup	Gross disease	4	14	2	2	10
	No gross disease	5	6	1	2	2
	Mastectomy	4	10	2	2	15
	BCS	4	9	1	1	2
	Hypofractionation	2	3	1	2	10
	Standard fractionation	5	16	2	1	0

Acute toxicity reported for 56 re-irradiations using CTCAE 3.0. Late toxicity reported for 47 patients (2 with bilateral disease) that had at least one course of re-irradiation.

### Initial radiotherapy

Radiation therapy was used for patients with breast conserving surgery or mastectomy and +/− positive lymph nodes. For those patients with breast conserving surgery, whole breast radiotherapy was delivered (50 Gy/25 fx or 42.5 Gy/16 fx). A boost was routinely recommended for patients < 50 years old or with high-risk factors. Boost irradiation (10 Gy-16 Gy) was administered to the tumour bed. Regional lymph nodes were also treated when positive. Patients with locally advanced breast cancer received neoadjuvant chemotherapy, followed by total mastectomy and then radiotherapy to the chest wall and lymph nodes (50 Gy in 25 fx + boost).

### Re-irradiation treatments

All patients with local recurrence were assessed by a multidisciplinary team with input from surgical, medical and radiation oncology. The time from the last day of original radiotherapy treatment and recurrence was calculated. Patients who previously received breast conserving surgery, but presented with recurrent disease were recommended for mastectomy.

Treatment volume included the whole breast/chest wall and regional lymph nodes. Field arrangements consisted of standard tangents and AP/PA fields to the supraclavicular lymph nodes. The most common dose/fractionation was 50 Gy/25 fx followed by a boost dose of 16 Gy in 8 fractions to the tumour cavity.

Patients with unresectable tumours were considered for definitive chemo-radiotherapy, eligible patients were treated to a dose of 65 Gy in 50 fx BID with concomitant cisplatin 20 mg/m^2^ weekly.

Radiotherapy was delivered to the breast/chest wall in 48 re-irradiation treatments including the axilla/supraclavicular nodes in 16 instances. Partial-breast radiotherapy was administered to 8 patients. The median dose was 50 Gy to the whole breast. 17 Patients received an additional boost of 16 Gy in 8 fx. Six patients were treated with a BID scheme. The accumulated 2 Gy equivalent (EQD2) to whole breast was 96.7 Gy (54.0-116.0) and 99.8 Gy (75.9-122.8) using α/β 10 and 3 respectively. [Supplementary Table 4].

### Systemic treatment

Patients were aggressively treated with systemic treatments according to local guidelines. Chemotherapy was offered to any patient fit for chemotherapy with triple negative disease or failing hormonal treatment. Patients included in this cohort had a median of 3 schemes used. 10 Patients had more than 5 different schemes. After local failure chemotherapy and/or hormonal therapy were recommended and a third course of radiation was used as a salvage treatment for failure for patients on systemic treatment.

### Intra-treatment assessment and post-treatment Follow-up

Patients were seen weekly by the treating physicians during treatment and assessed for radiation-related symptoms. Acute toxicity was graded using Common Terminology Criteria for Adverse Events (CTCAE) v3.0. Follow-up time started from the last day of radiotherapy. Patients were followed up with clinical visits at least every 3 months for the first 2 years, then every 6 months until 5 years, and then annually. Patients with breast conserving surgery had FU mammograms annually. Patient results were recorded in the electronic medical record at the time of medical and radiological consults.

### Statistical analysis

For survival data, three parameters were analyzed: survival without local recurrence (SWLR), survival without distant failure (SWDF) and overall survival (OS). The time of SWLR was measured from the last day of the retreatment to the day of local failure, and defined as any clinical progression of the disease in the treated area (breast/chest wall/ regional lymph nodes). The SWDF was defined as the time interval from the last day of the original radiotherapy treatment and the first evidence of systemic failure. The OS was calculated from the last day of retreatment and death from any cause. The OS, SWDF. SWLR, were analyzed globally and according to exploratory subgroups. Kaplan-Meier methodology was used and log rank in order to compare curves. We used a Cox proportional hazard regression model for multivariate analysis. Analysis was performed using IBM SPSS v19 (IBM, Chicago, USA).

## SUPPLEMENTARY MATERIAL FIGURES AND TABLES


