# Supplementary material for: Re-irradiation for locally recurrent refractory breast cancer
Source: Oncotarget. 2015 Oct 8;6(33):35051–62. doi: 10.18632/oncotarget.6036 (PMC4741508; doi:10.18632/oncotarget.6036)
Supplement: Supplementary file 1 [file oncotarget-06-35051-s001.pdf]

Re-irradiation for locally recurrent refractory breast cancer

Supplementary Material

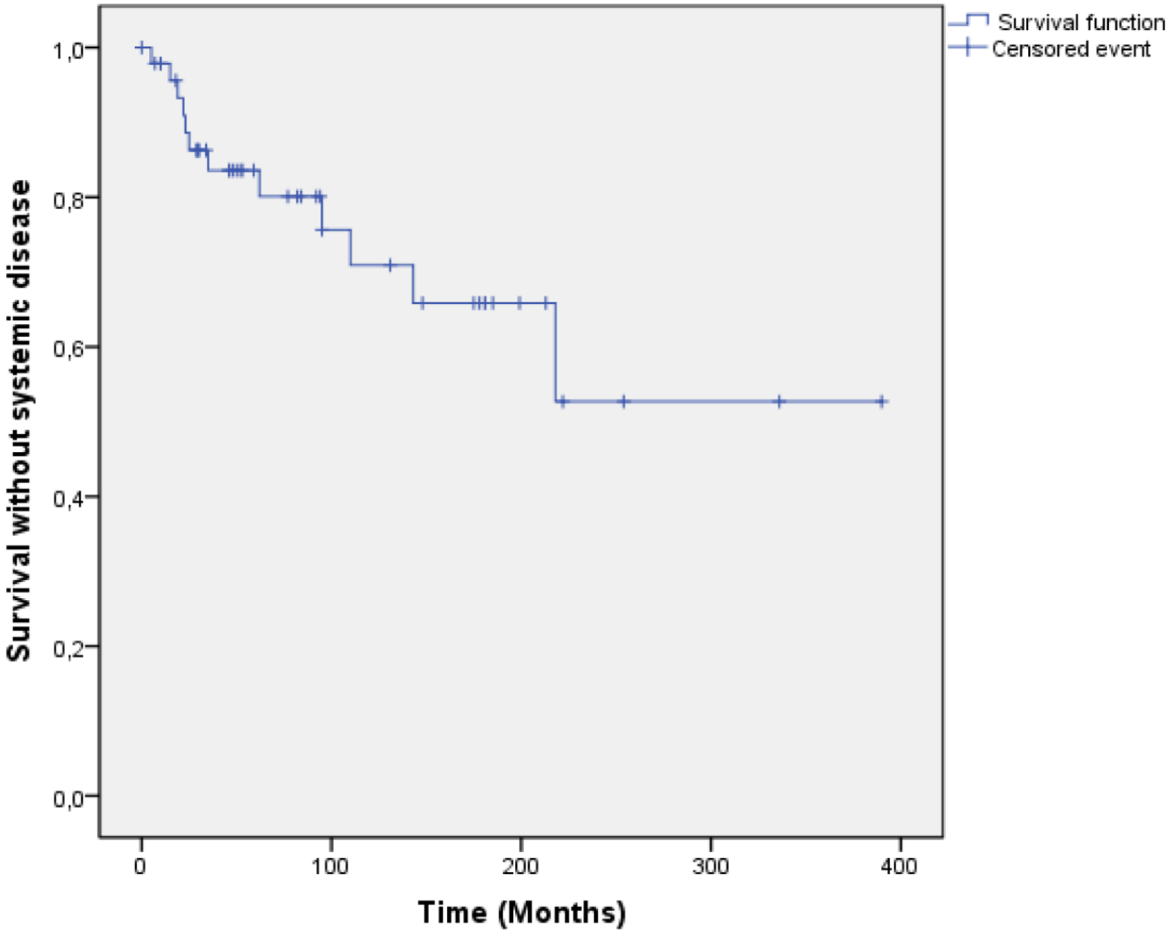

Supplementary Figure 1: Survival without systemic disease

Footnote:

Survival without systemic progression since first radiotherapy treatment to the first sign of systemic progression.

A

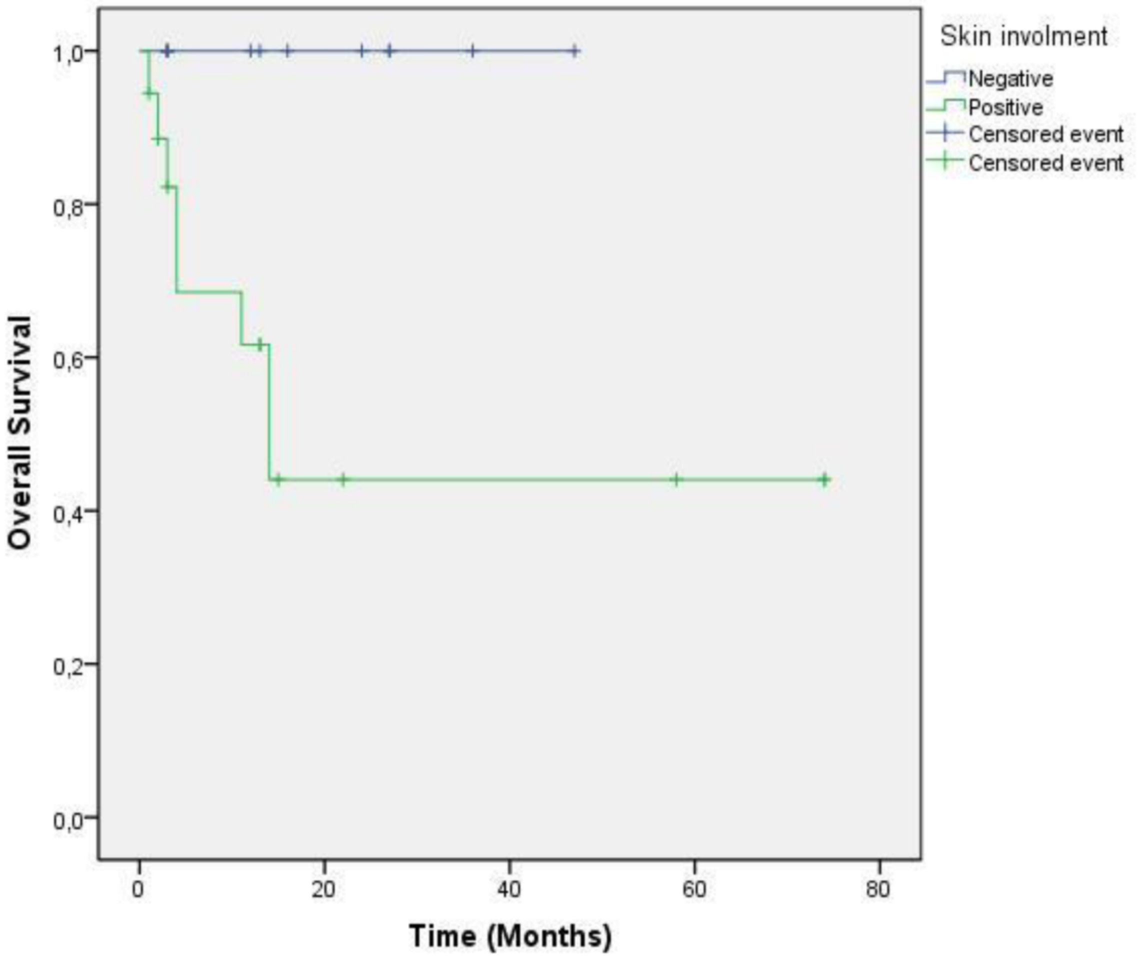

Supplementary Figure 2a; Overall survival according to skin involvement

Footnote:

- With skin involvement (green)
- Without skin involvement (blue)

p=0.013

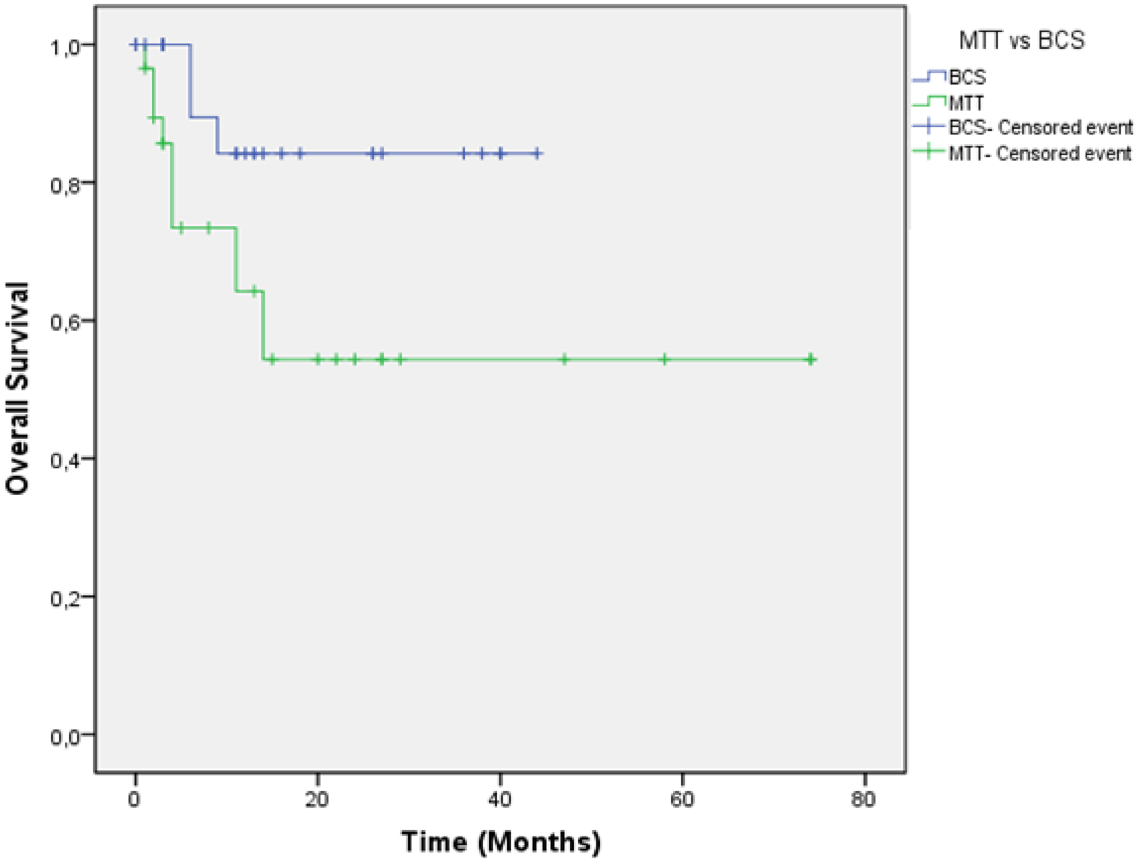

Supplementary Figure 2b: Overall Survival according to type of surgery Mastectomy vs Breast conserving surgery

Footnote:

Breast conserving surgery (blue)

Mastectomy (green)

p= 0.043

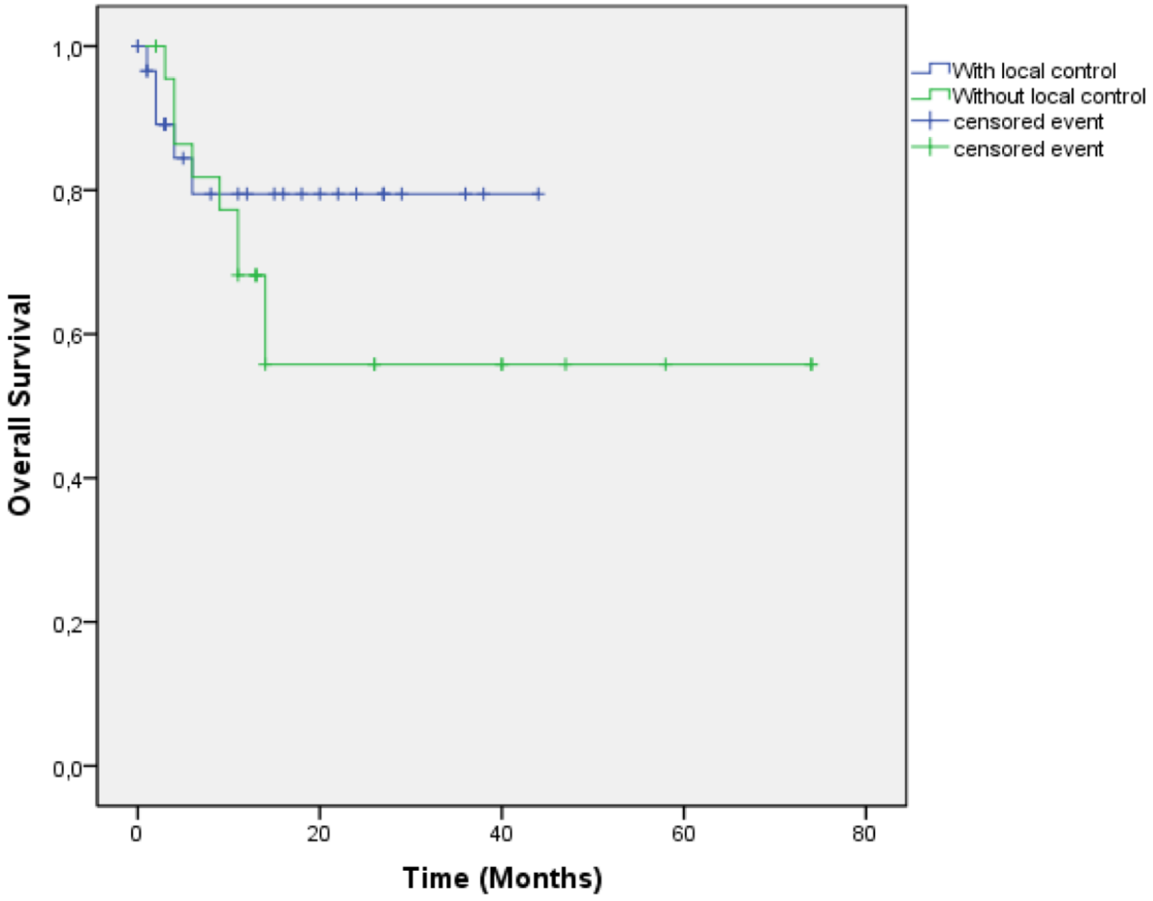

Supplementary Figure 3: Overall Survival according to local control

Footnote:

With local control (blue)

Without local control (green)

p= NS (non significant).

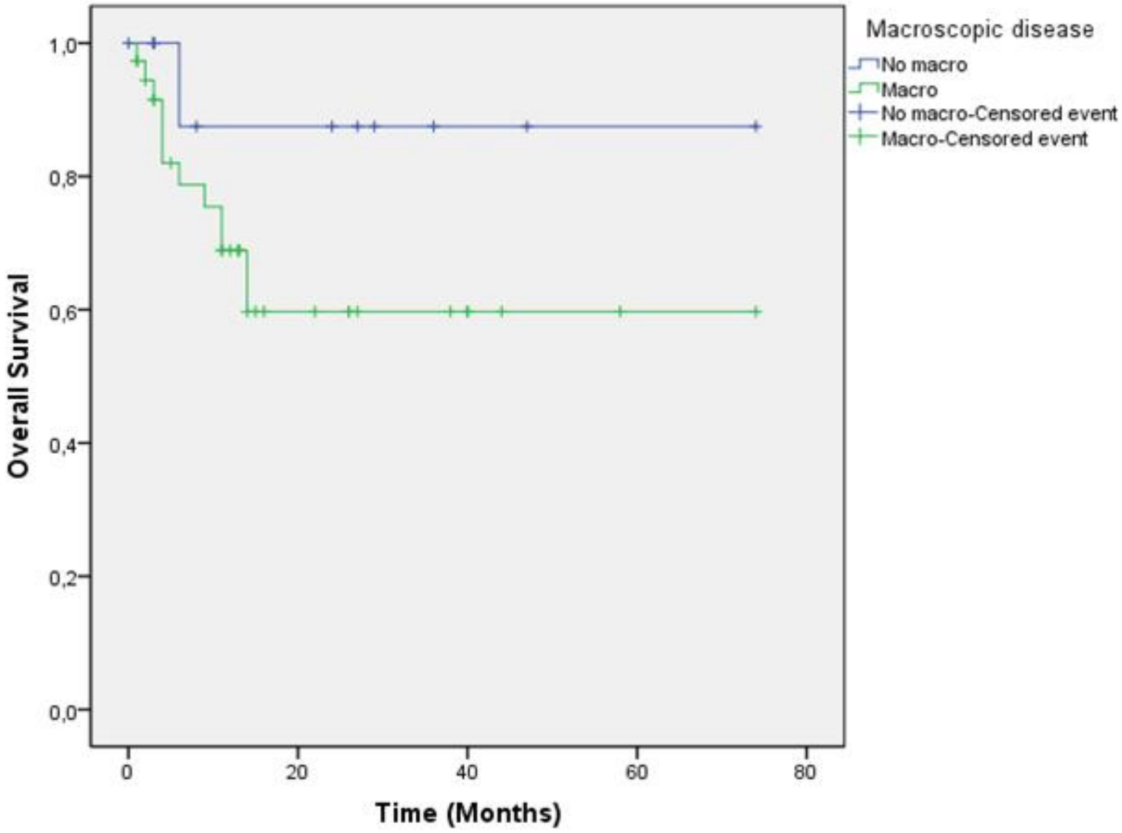

Supplementary Figure 4a: Overall Survival according to presence of macroscopic disease

Footnote:

No Macroscopic disease (blue)

Macroscopic Disease (green)

p= 0.172 NS(non significant).

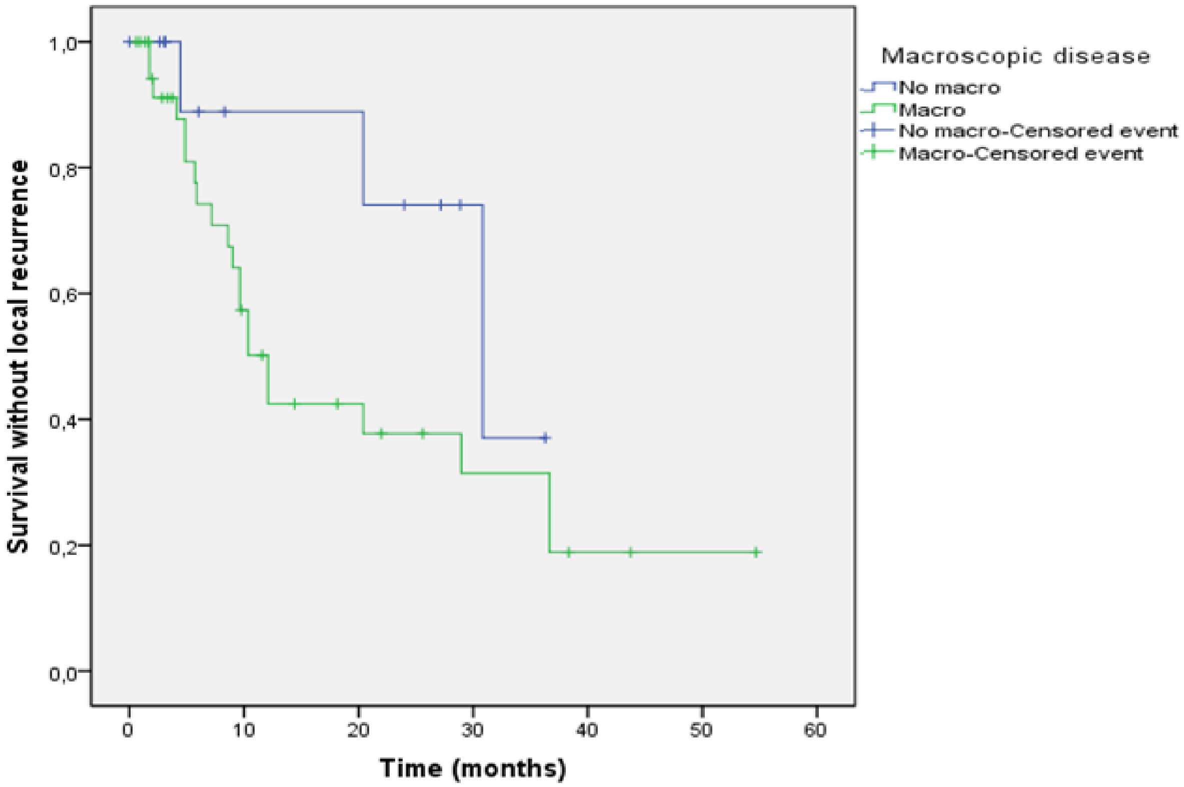

Supplementary Figure 4b: Survival without local recurrence according to presence of macroscopic disease

Footnote:

No Macroscopic disease (blue)

Macroscopic Disease (green)

p= 0.146 NS(non significant).

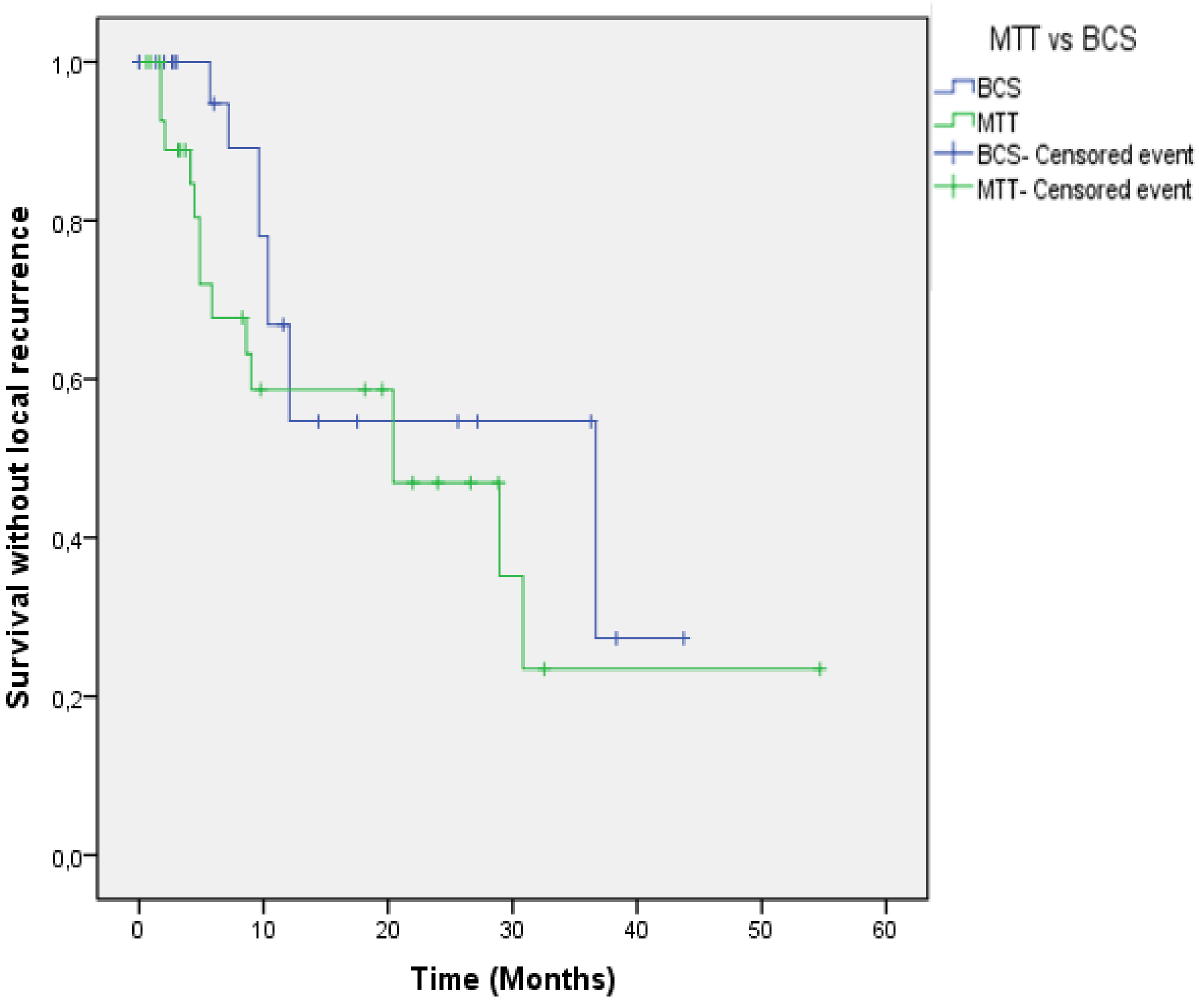

Supplementary Figure 5: Survival without local recurrence according to type of surgery MTT vs BCS

Footnote:

Breast conserving Surgery (BCS) (blue)

Mastectomy (MTT) (green)

p= NS (non significant).

Supplementary Table 1
1st treatment characteristics:

Pt	Surgery	Size (cm)	ER	LN Status	Dose WBRT (Gy)	Nº Fx	Boost Dose (Gy)	Nº Fx	LN Area Treatment
1	BCS	2	(-)	(-)	50.0	25	0	N/A	No
2	MRM	3	(-)	(-)	50.0	25	0	N/A	No
3	BCS	1.9	Unknown	(-)	50.0	25	0	N/A	No
4	MRM	12.6	(+)	(+)	50.0	25	16.0	8	Yes
5	BCS	1.9	(+)	(-)	Unknown	Unknown	Unknown	N/A	Unknown
6	MRM	9	(-)	Unknown	50.0	25	0	N/A	Yes
7	MRM	1.7	(+)	Unknown	50.0	25	0	N/A	Unknown
8	BCS	1.5	(+)	(+)	50.4	28	10.0	10	Yes
9	BCS	2	Unknown	Unknown	50.0	25	0	N/A	No
10	BCS	1.7	(+)	(-)	50.0	25	0	N/A	No
11	MRM	7	(+)	(+)	50.0	25	0	N/A	Yes
12	BCS	Unknown	Unknown	Unknown	50.0	25	0	N/A	No
13	MRM	Unknown	(+)	(-)	50.0	20	0	N/A	Yes
14	BCS	0.3	(+)	(-)	42.5	16	16.0	8	No
15	MRM	1.8	(+)	(+)	50.0	25	0	N/A	No
16	BCS	2.3	(-)	(-)	42.5	16	12.5	5	No
17	MRM	7	(+)	(+)	50.0	25	0	N/A	Yes
18	BCS	4.4	(+)	(+)	50.0	25	16.0	8	Yes
19	BCS	4	Unknown	(+)	50.0	25	16.0	8	Yes
20	MRM	2.5	(+)	(+)	50.0	25	16.0	8	Yes
21	BCS	0.75	(+)	(-)	42.5	16	0	N/A	No
22	MRM	2.5	(+)	(+)	50.0	25	0	N/A	No
23	BCS	2.3	(+)	(-)	42.5	16	0	N/A	No
24	MRM	4	(+)	(-)	50.0	25	0	N/A	Yes
25	BCS	2.5	(-)	(-)	42.5	16	0	N/A	No
26	BCS	2.9	(-)	(+)	50.0	25	16.0	8	Yes
27	BCS	1.8	(+)	(+)	50.0	25	16.0	8	Yes
28	MRM	Unknown	(-)	Unknown	50.0	25	10.0	5	Yes
29	MRM	19	(-)	(+)	65.0	50	0	N/A	Yes
30	BCS	3.1	(-)	(+)	50.0	25	0	N/A	Yes
31	BCS	Unknown	(+)	Unknown	42.5	16	12.5	5	Unknown
32	MRM	12	(+)	(+)	50.0	25	0	N/A	Yes
33	MRM	8	(-)	(+)	50.0	25	16.0	8	Yes

34	BCS	1.7	(+)	(-)	48.0	24	15.0	5	No
35	NO	Unknown	(+)	(+)	50.0	25	20.0	10	Yes
36	BCS	Unknown	(-)	(-)	42.5	16	10.0	5	No
37	NO	Unknown	(-)	Unknown	65.0	50	0	N/A	Yes
38	BCS	3	(-)	(+)	50.0	25	16.0	8	Yes
39	MRM	3.8	(+)	(+)	50.0	25	0	N/A	Yes
40	MRM	Unknown	(-)	(+)	50.0	25	0	N/A	Yes
41	BCS	2.5	(-)	(+)	42.5	16	12.5	5	No
42	BCS	3.2	(+)	(+)	42.5	16	12.5	5	Unknown
43	BCS	Unknown	Unknown	Unknown	Unknown	Unknown	Unknown	Unknown	No
44	MRM	15	(-)	(+)	6.0*	3*	0	N/A	No
45	MRM	2.3	(+)	(+)	Unknown	Unknown	Unknown	Unknown	Yes
46	BCS	1.5	(+)	(+)	50.0	25	16.0	8	No
47	BCS	Unknown	(-)	(+)	50.0	25	10.0	10	Unknown
48	MRM	3	(-)	(+)	45.0	25	20.0	10	Yes
49	MRM	1.5	(+)	(+)	50.0	25	0	N/A	No

BCS: Breast conserving surgery, ER Estrogen receptor, LN: lymph node, MRM: modified radical mastectomy, N/A: Non applicable, WBRT: whole breast radiotherapy * Patient 44 suspended radiotherapy after 6 Gy.

Supplementary Table 2
2nd Retreatment patient characteristics

Pt	Age (years)	Time to 2nd recurrence (months)	1st treatment WBRT Dose EQD2 Dose (α/β = 3)	2nd Treatment WBRT Dose EQD2 Dose (α/β = 3)	3rd Treatment WBRT Dose EQD2 Dose (α/β = 3)	Total dose* (cGy) EQD2 treatments α/β = 3
6	55	2	50.0	56.0	81.6	203.6 (1)
31	73	9	48.1	50.0	54.0	171.1(2)
23	89	11	48.1	50.0	67.5	181.6 (3)
36	67	12	48.1	55.9	27.5	141.5 (4)
8	49	12	48.4	32.4	11.0	101.8(5)
18	43	37	50.0	36.0	36.0	138.0 (6)
13	72	48	55.0	28.0	54.0	137.0

*Total dose equivalent to the sum of dose delivered in all three treatments. Including Boost (1); 16 Gy boost in first treatment, (2): 13.75 Gy boost on first treatment and 16 Gy boost on second treatment, (3) 16 Gy Boost on 2nd Treatment, (4) 10 Gy boost on first treatment. (5): 10 Gy boost on first treatment. (6) 16 Gy Boost on first treatment.

Supplementary Table 3

Supplementary table: Systemic treatments, reason to retreat and evolution

Pt	HT	Systemic treatment	Reason to treat	Evolution
1	Yes	FEC-TH, clinical trial T-DM1 +/- Pertuzumab, Femara, Vinorelbine and Herceptin, Zoladex	Recurrent local progression under systemic treatment	Stable disease
2	No	Taxol Herceptin, Vinorelbine plus Herceptin	Partial breast re-irradiation	Acute erythema, fibrosis, telangiectasia, stable disease
3	No	FEC-T, Cisplatin, Gemcitabine, Capecitabine, vinorelbin clinical trial Eribulin, Etoposide	Progressive pain and swelling from plexus involvement	Extensive fibrosis, initial response, then local and systemic progression
4	Yes	TC, TMX, Zoladex Exemestane	Recurrence short after original treatment	Radiation dermatitis, no long term toxicity
5	Yes	Herceptin, Vinorelbine Herceptin	Multiple low cervical + SCV LN	Moist desquamation, fibrosis, clinical response stable, progression with lung metastases
6	Yes	FEC100, Taxotere Cyclophosphamide, Capecitabine, Paclitaxel, Vinorelbine	Large mass, brachial plexopathy	Acute erythema, fibrosis, systemic progression
7	Yes	FECT, Letrozole, Abraxane, Zoladex, Capectiabine, Cyclophosphamide and Methotrexate, Vinorelbine	Recurrent nodules in skin and 6 cm axilar mass	Initial response, local and systemic failure
8	Yes	FEC, Arimidex, Clondronate, Exemestane, Capecitabine, Taxotere, Faslodex, Vinorelbine, Etoposide, Cyclophosphamide, Metrotrexate	Large mass compromising brachial plexus	Initial response, radiation dermatitis, fibrosis, then local progression
9	Yes	FECT, Gemcitabine Cisplatin, Cisplatin concomitant RT	Progression under chemotherapy	No toxicity, progression borders of the field
10	Yes	ACT, Letrozole	Multiple risk factors for recurrence	Radiation myositis, hyper pigmentation
11	No	ACT, Cisplatinum Gemcitabine, Capecitabine	Large recurrent mass in chest wall	Moist desquamation, no long term toxicity
12	Yes	Taxol, Astrin, Abraxane, Avastin, Gemcitabine, Cisplatin, Zoladex, Ixempra, Xeloda	Bleeding mass	No acute toxicity, no long term FU
13	Unknown	Unknown	Large mass involving brachial plexus	Clinical response less numbness and tumour size, no long term toxicity

14	No	Dose dense ACT, Cisplatin Gemcitabine, Taxotere with RT concomitant	Ulceration and progression under chemotherapy	Complete clinical response, systemic progression in lung, PE and death
15	Yes	ACT, Xeloda, Paclitaxel	Isolated recurrence in low neck and SCV LN	Radiation neumonitis, progression mediastinum and SCV LN
16	No	Cisplatin	Ulcerative bleeding, bad odor	Prominent moist desquamation, radiation neumonitis, fibrosis prominent, telangiectasia prominent
17	No	Unknown	Multiple risk factors	Acute erythema, moist desquamation
18	Yes	Tamoxifen, Arimidex, FEC 50	Unknown	Clinical response and then progression
19	Yes	Neoadjuvant ACT, Xeloda/Capecitabine but abandoned for alternative medicine, Carboplatinum, Gemcitabine, Xeloda, TMX	Fungating mass, ulcerated skin	Control of disease, marginal recurrence treated with RT
20	Yes	FEC, Letrozole	Oligo metastasic disease, progressing locally	Hyper pigmentation and erythema, stable disease in treated area
21	Yes	FEC, Arimidex, Clondronate, Exemestane, Capecitabine, Taxotere, Faslodex, Vinorelbine, Etoposide, Cyclophosphamide, Methotrexate	Progression under chemotherapy	Clinical response, acute dermatitis, telangiectasia
22	No	FEC-T, Xeloda, Gemcitabine/Carboplatinum, PARP inhibitor trial, Vinorelbine	Massive chest wall recurrence, progressing on chemotherapy	Progression contra lateral breast, suspended treatment for skin reaction, no response
23	No	FEC T Herceptin	Local recurrence, oligo metastasis	Erythema, hypo pigmentation, fibrosis, systemic progression, brain metastasis
24	No	Weekly Paclitaxel and Avastin, Capecitabine, Pamidronate, Cisplatin/Gemcitabine	Original rejected adjuvant treatment wirh local recurrence	Moist desquamation, systemic progression on lung and liver, death
25	No	ACT Herceptin	Skin involvement, fungating mass, progression under chemotherapy	Good response, healing of fungating mass, significant moist desquamation requiring dressing, then recurrence started on chemotherapy, death at home
26	Yes	ACT, Cisplatin concomitant with RT, Letrozole, Capecitabine concomitant,	Immediate local recurrence	Initial response, but local progression

		Vinorelbine		
27	Yes	Femara, Taxol Herceptin, AC, Capecitabine, Lapatinib, Cisplatin weekly	Large mass, skin involvement, refractory to chemotherapy	Clinical response, no toxicity reported
28	Yes	CMF, A T, Letrozole, Clodronate to Pamidronate, Xeloda, Navelbine, Vinorelbine, Abraxane, Etoposide, Gemcitabine, Exemestane	Large mass in axila, progression under chemotherapy	Moist desquamation, dryness skin, initial response, then recurrence and local and systemic progression
29	No	FEC 100,Taxotere	Multiple risk factors	Telangiectasia, fibrosis
30	Yes	Pamidronate	Neurological pain	Complete clinical response, fibrosis, systemic progression
31	Yes	TMX, Letrozole	Unknown	No recurrence
32	No	T-DM1 trial, Pamidronate, Gemcitabine T	Painful chest wall, para-sternal mass on T-DM1 trial	Moist desquamation, clinical response, hypo pigmentation, telangiectasia, fibrosis
33	No	AC, Cisplatin concomitant RT, clodronate, FEC, Capecitabine, Cisplatin, Gemcitabine, Eribulin	Fungating bleeding mass, progression under chemotherapy	Clinical response and recurrence contralateral
34	Yes	Unknown	2nd partial breast radiotherapy	Acute erythema, fibrosis, no evidence of disease
35	Yes	FEC TH, Aromasin	Partial breast re-irradiation	Moist desquamation, fibrosis, telangiectasia, lymphedema
36	No	ACT	Multiple risk factor	Lymphedema, no evidence of disease
37	No	Cisplatin concomitant, Pamidronate, Cisplatin and Gemcitabine, Eribulin	Fungating mass refractory to chemotherapy	Clinical response and subsequent local and systemic progression
38	No	CMF	Unknown	Unknown
39	No	FECT, T, Abraxane trial, Csplatin, Cyclophosphamide, Methotrexate	Large fungating mass, refractory to chemotherapy	Moist desquamation, clinical response, then local progression
40	Yes	Arimidex, Tamoxifen, mTOR inhibitor trial, FraCMP Trial, Everolimus clinical trial, Cyclophosphamide and Methotrexate, Megace	Growing under systemic treatment, Significant pain	Moist desquamation entire chest wall, telangiectasia G2, fibrosis G2, necrotic skin complete resolved 3 months, Clinical response but local progression
41	Yes	Femara, Aromasin, Xeloda, Faslodex, Vinorelbine, Methotrexate and Cyclophosphamide	2nd local recurrence with surgery, progression	Good response healing of fungating mass, necrosis, fibrosis, but then recurrence starting

			under systemic treatment	Chemotherapy
42	Yes	Letrozole	Mass axila with pain plexus involvement, neurological deficit and lymphedema	Clinical response, less pain and more movement, local failure, persistent Lymph edema
43	Yes	TMX, Femara, Taxol, Exemestane, Capecitabine	Recurrent mass in axila involving brachial plexus	Control of disease, moist desquamation, fibrosis axila, lymph edema
44	Yes	Unknown	Fungating mass	Moist desquamation, Mediastinum progression
45	Unknown	Unknown	Massive recurrence involving the sternum	No long term FU
46	Yes	CMF	Multiple Risk factors	hyper pigmentation
47	Yes	TMX	Recurrent 20 years after different histology new primary	Good response, Only Erytema
48	Unknown	Unknown	10 yr different histology, 2nd primary	No acute toxicity no log term FU
49	Yes	Arimidex, Octeotride (CLL)	Axilar recurrent and growing mass under systemic treatment	Moist desquamation, hypo pigmentation, field margin and axila progression

ACT: adriamycin cyclophosphamide, taxol; CLL: Chronic lymphocytic leukemia; CMF: cyclophosphamide methotrexate fluorouracil; FECT: fluorouracil, epirubicin cyclophosphamide Docetaxel; H: herceptin, LN: Lymphonode; SCV: Supraclavicular; TMX: Tamoxifen

Supplementary Table 4:

	1st Treatment								
**Pt**	**Dose WBRT (Gy)**	**Nº Fx**	**Boost Dose (Gy)**	**Nº Fx**	**Dose WBRT (Gy)**	**Nº Fx**	**Boost Dose (Gy)**	**Nº Fx**	**Total WBRT dose* (cGy) EQD2 treatments α/β = 3**
1	50.0	25	0	N/A	60.0	15	0	N/A	122.0
2*	50.0	25	0	N/A	60.0	15	0	N/A	122.0
3	50.0	25	0	N/A	50.0	20	20.0	10	105.0
4	50.0	25	16.0	8	25.0	10	0	N/A	77.50
5	Unknown	Unknown	Unknown	N/A	50.0	25	16.0	8	
6	50.0	25	0	N/A	40.0	10	16.0	8	106.0
7	50.0	25	0	N/A	66.0	33	0	N/A	116.0
8	50.4	28	10.0	10	36.0	24	0	N/A	80.8
9	50.0	25	0	N/A	Unkown	Unkown	Unkown	N/A	Unkownn
10	50.0	25	0	N/A	50.0	25	20.0	10	100.0
11	50.0	25	0	N/A	27.0	15	38.0	10	75.9
12	50.0	25	0	N/A	36.0	12	0	N/A	93.2
13	50.0	20	0	N/A	20.0	5	0	N/A	83.0
14	42.5	16	16.0	8	50.0	25	10.0	5	98.1
15	50.0	25	0	N/A	60.0	30	0	N/A	110.0
16	42.5	16	12.5	5	60.0	30	0	N/A	108.1
17	50.0	25	0	N/A	45.0	25	0	N/A	93.2
18	50.0	25	16.0	8	30.0	10	0	N/A	86.0
19	50.0	25	16.0	8	50.0	25	0	N/A	100.0
20	50.0	25	16.0	8	45.0	25	15.0	10	93.2
21	42.5	16	0	N/A	50.0	25	0	N/A	98.1
22	50.0	25	0	N/A	52.0	13	0	N/A	122.8
23	42.5	16	0	N/A	50.0	25	16.0	8	98.1
24	50.0	25	0	N/A	45.0	10	0	N/A	117.5
25	42.5	16	0	N/A	50.0	25	20.0	10	98.1
26	50.0	25	16.0	8	50.0	25	0	N/A	100.0
27	50.0	25	16.0	8	45.0	25	21.0	2.6	93.2
28	50.0	25	10.0	5	45.0	25	0	N/A	93.2
29	65.0	50	0	N/A	45.0	15	0	N/A	109.9
30	50.0	25	0	N/A	40.0	10	0	N/A	106.0
31	42.5	16	12.5	5	50.0	25	16.0	8	98.1
32	50.0	25	0	N/A	50.0.	25	16.0	8	100.0
33	50.0	25	16.0	8	37.7	29	11.7	2.3	82.4

34	48.0	24	15.0	5	50.0	25	0	N/A	98.0
35	50.0	25	20.0	10	40.0	10	0	N/A	106.0
36	42.5	16	10.0	5	65.0	50	0	N/A	104.0
37	65.0	50	0	N/A	25.0	10	0	N/A	83.4
38	50.0	25	16.0	8	50.0	25	16.0	8	100.0
39	50.0	25	0	N/A	65.0	50	0	N/A	105.9
40	50.0	25	0	N/A	65.0	50	0	N/A	105.9
41	42.5	16	12.5	5	40.0	10	0	N/A	104.1
42	42.5	16	12.5	5	35.0	5	0	N/A	118.1
43	Unknown	Unknown	Unknown	Unknown	50.0	25	0	N/A	Unkown
44	6.0*	3*	0	N/A	32.0	4	0	N/A	76.4
45	Unknown	Unknown	Unknown	Unknown	66.0	33	0	N/A	Unkown
46	50.0	25	16.0	8	50.0	25	16.0	8	100.0
47	50.0	25	10.0	10	65.0	50	16.0	8	105.9
48	45.0	25	20.0	10	48.7	20	0	N/A	96.1
49	50.0	25	0	N/A	45.0	25	0	N/A	93.2

WBRT: Whole breast Radiotherapy dose (Not including the boost) *Total dose equivalent to the sum of dose delivered original and first re-irradiation treatments.
